# Psychophysical assessment of color vision with the Cambridge Color Vision Test in unilateral functional amblyopia

**DOI:** 10.5935/0004-2749.2023-0263

**Published:** 2024-12-26

**Authors:** Roberta Melissa Benetti Zagui, Leonardo Dutra Henriques, Marcelo Fernandes Costa

**Affiliations:** 1 Research Nucleus in Neuroscience and Behavior and Applied Neuroscience, Universidade de São Paulo, São Paulo, SP, Brazil; 2 Department of Ophthalmology, Faculdade de Medicina, Universidade de São Paulo, São Paulo, SP, Brazil; 3 Department of Experimental Psychology, Institute of Psychology, Universidade de São Paulo, São Paulo, SP, Brazil

**Keywords:** Amblyopia, Anisometropia, Color vision, Strabismus, Vision disorders, Visual acuity

## Abstract

**Purpose:**

Amblyopia is a cortical neurological disorder caused by abnormal visual experiences during the critical period for visual development. Recent works have shown that, in addition to the well-known visual alterations, such as changes in visual acuity, several perceptual aspects of vision are affected. This study aims to analyze and compare the effects of different types of amblyopia on visual color processing and determine whether these effects are correlated with visual acuity.

**Methods:**

Our study sample comprised 42 amblyopic individuals, aged 7-40 years, (strabismus, n=16; anisometropia, n=18; and mixed-cause, n=8) and 33 age-matched controls. Color vision was tested by measuring the chromaticity threshold of each patient on the protan, deutan, and tritan axes using version 02 of the Cambridge Color Test. Spatial stimulation cues were eliminated using spatial noise and luminance.

**Results:**

The color discrimination thresholds on the protan, deutan, and tritan axes were similar between control participants and amblyopic patients (p>0.05). There was no correlation between VA values and color thresholds (p>0.05).

**Conclusion:**

Patients with amblyopia have normal color vision in contexts that include luminance and spatial noise. Our results may be indicative of independent neural pathways for spatial and chromatic visual processing.

## INTRODUCTION

Amblyopia is a cortical neurological disorder caused by an abnormal visual experience during the critical period of visual development^(1)^. leading to a significant intraocular difference in visual acuity breaking the single binocular vision^(2)^. Its severity is often determined considering the age. On this basis, amblyopia may be classed as mild (a logarithmic measure of angle of resolution [logMAR] of 0.2-to 0.4), moderate (0.4-0.7 logMAR), or severe (>0.7 logMAR). Studies on amblyopia have found the common alterations in visual functions characteristic of this condition to be changes in VA, contrast sensitivity (CS), and stereopsis. However, a recent study has shown that different types of amblyopia lead to different visual deficits and numerous local and global perceptual functions of vision may be affected^(3)^. Among functions affected there is a current interest in color vision in amblyopia^(4)^.

Color vision in amblyopia has been poorly studied. This is somewhat surprising as color vision is processed by the parvocellular (PC) pathway, which is known to be affected by amblyopia. It is also a visual function that follows a progressive development curve until the end of adolescence (between 18-20 years old)^(5^,^6)^, coinciding with the plasticity of the visual system and corresponding risk of amblyopia. The few works on color vision in amblyopia have either found no abnormalities in color vision or inconsistent results^(7^,^8^,^9)^.

Red-green chromatic stimuli are preferentially processed via the PC pathway; whereas blue-yellow stimuli are processed via the koniocellular (KC) pathway, which is little studied in amblyopia^(10)^. New research has identified a loss of the normal projections of the foveal cones in eyes affected by amblyopia, with a direct impact on the PC pathway. It has also demonstrated alterations in the chromatic and achromatic pathways, with the chromatic pathway showing greater alterations in response to complex chromatic stimuli^(11)^. A study using chromatic evoked visual potential suggests markedly worse chromatic sensitivity in amblyopic patients, both in the affected and unaffected eye^(12)^.

The methodology used in studies of color vision in amblyopia has been heterogeneous. A study in 2006 evaluated color vision in amblyopia using screening tests for congenital and deep color-vision defects such as the Ishihara and Hardy-Rand-Rittler (HRR) tests, which have relatively low sensitivity compared to other available tests. The study also used color appearance ordering tests such as the Farnsworth Munsell 100 Hue (FM-100) Test, the Farnsworth D-15 Color Blind Test, and the Roth 28-Hue Test, which have little diagnostic value^(13)^. A more recent study evaluated sensitivity to chromatic contrast with stimuli sinusoidal grids and checkerboards as the stimuli^(14)^. However, these have an intrinsic spatial component that could influence the cortical response. To properly assess color vision in amblyopia, the variable measured (i.e., color vision) must be sufficiently separated from other visual variables, such as spatial components of the stimulus.

Given the paucity and limitations of existing research, a thorough and stringent investigation the effects of amblyopia on color vision is yet to be conducted. More sensitive computerized psychophysical color-vision assessments such as Mollon and Reffin’s Cambridge Color Test (CCT)^(15)^, have recently been used in patients with reduced binocular VA; although no deficits on any of the color confusion axes were found^(16)^. The CCT uses pseudoisochromatic plates as stimuli and follows a psychophysical progression through chromatic steps that change according to the participant’s responses to allow for rigorous threshold estimation. The CCT simultaneously tests the three confusion lines of each cone type (visible light with short, medium, and long wavelengths). This allows a more refined analysis of the visual pathways involved in chromatic processing^(17^,^18^,^19)^.

This study aims to analyze and compare the effects of the different types of residual amblyopia on visual processing of color and to identify any correlations between alterations in color vision and VA. We use the CCT to achieve this, both for its sensitivity to color-vision disparities and for its provision of chromatic discriminative stimuli without spatial components. Spatial vision can be regarded as the binocular combination of VA and luminance CS. Therefore, we hypothesize that assessment of color vision in amblyopic patients using stimuli without local spatial components, as borders should not be affected in amblyopia.

## METHODS

### Participants

Participants were selected from volunteers aged 1040 with a childhood history of treatment for amblyopia. Individuals in the same age range with no history of eye disease were selected for inclusion in the control group. All volunteers and the parents of those under 18 agreed to study participation and signed a form attesting to their free and informed consent. The study was conducted in accordance with the tenets of the 2013 revision of the Declaration of Helsinki and was approved by the Plataforma Brasil Research Ethics Committee (protocol no. 66767317.5.0000.5561).

Each participant underwent a complete ophthalmologic examination. This included VA measurement using the early treatment diabetic retinopathy study chart (logMAR), determination of the dominant eye (DE) using the Dolman Distance Hole-in-the-Card Test^(20)^, dynamic and static refraction under cycloplegia (with an appropriate optical prescription if necessary), complete evaluation of extrinsic ocular motility and binocular vision using the Titmus Test and the Four-Diopter Test for ocular deviation measurement and stereoscopic VA measurement, biomicroscopy, testing of pupillary reflexes, ectoscopic analysis, and retinal mapping under pupillary dilation. All patients and controls underwent the experimental tests in a single examination session. Each test was monocular and we began with the DE in both the amblyopic and control participants. The inclusion criteria for the control group were normal VA of optotypes (<0.0 logMAR); stereopsis (<40 arc seconds), with optical correction if required; no permanent or intermittent strabismus or binocular disturbances; no other ocular pathologies; and free and informed consent to study participation. The exclusion criteria were high ametropias (a spherical equivalent >12 diopters), neurological or cognitive deficits, and the use of drugs that affect the central nervous system.

The inclusion criteria for the amblyopic group were reduced optotype best-corrected VA of 1 octave or an interocular vision difference >0.1 logMar, previous completed treatment of amblyopia with stable results for more than 6 months, strabismus (also known as hete-rotropia) with or without previous surgical treatment, a significant interocular difference in refractive error (above 1.5 diopters spherical or 1.0 diopter cylindrical), no other ocular pathologies, and free and informed consent to study participation. The exclusion criteria were deprivation amblyopia, previous eye surgery for any reason other than strabismus correction, VA <0.8 logMar or >0.1 logMAR, high ametropia (spherical equivalent >12 diopters); neurological or cognitive deficits, and the use of drugs that affect the central nervous system.

### Stimuli and equipment

The color discrimination ability of all participants was evaluated using the commercial version of the CCT v.2.0, Cambridge Research Instruments, Rochester, UK)^(15)^, with a VSG 2/5 graphics card (Cambridge Research Instruments). The stimuli were generated on a, Sony FD Trinitron model GDM-F500T9 high-resolution color monitor, which was calibrated using a Chroma Meter CS-100A (Konica Minolta Inc., Japan).

**Figure 1:**
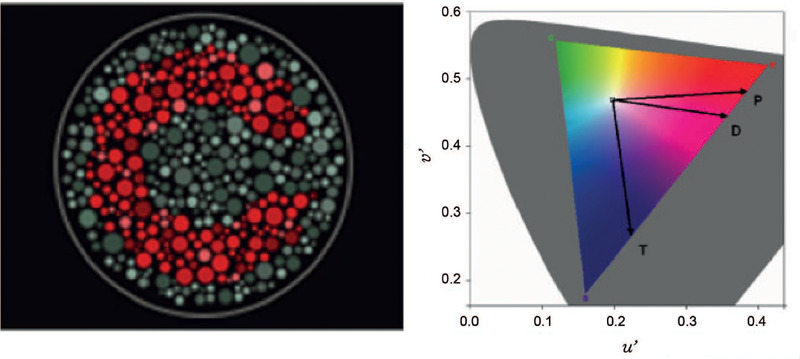
Illustrations of the stimuli used in the Cambridge Color Test to assess color vision. The image on the left is a pseudoisochromatic stimulus containing circles of different sizes and six luminance levels. The image on the right is a Landolt letter “C” with chromaticity that differs from that of the background. The opening of the C can face one of four directions: up, down, left, or right.

The stimulus was a pseudoisochromatic matrix of circles in which the target is a Landolt “C” that differs in chromaticity from the background, which is centered on white (coordinates 0.1977, 0.4689 in u’v’ units of the CIE 1976 color space). Seen at 3 meters, the size of the outer circumference of the C of Landolt corresponds to a visual angle of 4.3° and the inner circumference to a visual angle of 2°. The opening of the letter C subtends 1° of visual angle at 3 meters distance (Figure 1).

The target and background consisting of circles of various sizes has six randomly distributed luminance levels between 8-18 cd/m². These two strategies ensure that the participant detects the target using only the chromaticity differences, eliminating spatial cues and preventing the use of contour artifacts, simultaneous contrast, or luminance differences.

### Procedure

In this study, we used the shorter version of the CCT, known as the Trivector version. The Landolt C target is presented facing one of four directions (randomly selected): top, bottom, right, or left, for 6 seconds. During this time, the participant must press one of four answer box buttons (CT6 – Cambridge Research Instruments), selecting the button that corresponds to the perceived opening direction of the letter “C”. Computerized controls allow the chromaticity differences between the background and the target to be dynamically adjusted according to each participant’s performance. The experimenter uses this to maintain a constant correctness level of 79.4%.

The target differs from the white-centered background across the three color confusion axes to test for protan, deutan, and tritan defects. These defects correspond to the cones for long, medium, and short wavelengths of light. The presentation of the three axes is randomized and, periodically, a control target is presented to assess the reliability of the answers given. At the start of the test, the targets presented are highly saturated (distance of 1100 u’ v’ units for each confusion axis). Using the psychophysical staircase methodology and the dynamic response-based target adjustment strategy described above, a threshold between the chromaticity of the target and that of the background is maintained. This threshold is the minimum chromaticity difference at which the participant can detect the orientation of the letter “C”. An adaptive psychophysical staircase was used to measure the chromaticity thresholds for each confusion axis. The first two reversals in chromaticity were adjusted to decrease the difference between the saturation of the target and the background by 50%, with further difference increases of 25% with each new image. To maintain the steady progression in difficulty using participant’s responses, subsequent reversals that increased the difference between target and background chromaticity were in 25% increments. Those that decreased the difference between the target and background were in increments of 12.5%. The small excursion was 0.200 u’ v’ units). Each participant evaluation included 11 reversals, and the participant’s chromaticity threshold was determined using the average value of the last six reversals.

### Data analysis

Statistical analysis was performed using Statistica, version 10.2 (Statsoft, Tulsa, USA) software. A full descriptive analysis was performed. The normality of the distributions of each variable was assessed using the Kolmogorov-Smirnov and Shapiro-Wilks tests, which were applied to both the data and their residual values. For comparison purposes, the nonamblyopic eyes (NAEs) of the patients in the experimental groups were compared with the DE of the control group participants. The amblyopic eyes (AEs) of the patients in the experimental groups were compared with the NDE of the control group participants. Data analysis consisted of comparisons between groups and correlational analysis of variables. The data were described as means and standard deviations. Between-group comparisons were performed using repeated measures analyses of variance (RM-ANOVA). Each participant’s DE and NDE were evaluated to check for the dependence of our measurements. The significance level was set at 5% (p£0.05). Post hoc analyses were performed using Fisher’s least significant difference test. Correlations between factors were identified using Pearson’s correlation coefficient.

Effect sizes are a crucial outcome of empirical research, as they inform the reader whether an intervention or experimental manipulation has had a measurable impact beyond zero or, when a clear effect occurs, how substantial it is^(21)^.

## RESULTS

The sample in this study consisted of 75 volunteers recruited from a private eye clinic. All participants were aged between 10-40 years. The control group incorporated 33 individuals with healthy eyes (15.6 ± 8.7 years) and the experimental group was comprised of the remaining 42 participants, all of whom were amblyopic (14.3 ± 5.5 years). Participant examinations were performed at the Vision Lab of the Psychology Institute of the University of São Paulo (USP). The sample included 50% of each sex in both groups. The mean ages for each amblyopic type were 16.3 (±6.2) years in the anisome-tropic group, 15.7 (±5.5) years in the strabismic group, and 11.7 (±3.1) years in the mixed group.

In the amblyopic group, there were 16 participant diagnosed with amblyopia due to manifest or residual ocular deviations, with or without previous surgical correction (strabismus), 18 with anisometropic amblyopia, and eight with mixed strabismic and anisometropic amblyopia.

The descriptive data of the groups are summarized in tables 1 and 2 for the amblyopic and controls, respectively. The effect sizes for the control group compared to all amblyopic patients was classified as very large (η^2^ = 0.840) based on the Cohen classification system updated by Sawilowsky^(22)^.

**Table 1:** Descriptive data of the patients with amblyopia in our study

Group	Age	Amblyopia	VA RE	VA LE	SVA	RX RE	RX LE	Deviation	4 PD TEST
**S**	9	LE	0.0	0.2	ABSENT	+ 1.50 -0.75 × 180	+1.50 -1.00 × 20	ET 40 PD	
**S**	33	LE	-0.1	0.5	ABSENT	+ 3.25 -1.25 × 180	+4.00 -1.50 × 170	M1CRLET	
**S**	8	LE	-0.1	0.4	ABSENT	+ 3.25 -0.50 × 180	+3.25	ET 20 PD	
**S**	10	RE	0.7	-0.1	ABSENT	+ 2.50	+2.50 -0.50 × 90	ET 10 PD	
**S**	7	LE	0.0	0.3	ABSENT	+ 0.75 -0.75 × 180	+0.75 -0.75 × 25	ET 30 PD	
**S**	18	RE	0.1	-0.1	ABSENT	-0.50 × 180	PLANO	ET 50 PD	
**S**	26	LE	0.0	0.7	ABSENT	+ 1.50 -0.75 × 155	+ 1.25 -0.50 × 65	ET 40 PD	
**S**	13	LE	0.1	0.3	60"	+ 6.00 -2.50 × 180	+6.00 -2.50 × 10	M1CRLET	
**S**	14	LE	-0.1	0.6	ABSENT	-1.50 -0.75 × 100	-2.00 -0.50 × 100	ET 60 PD	
**S**	12	LE	-0.2	0.5	ABSENT	+ 0.50 -0.50 × 155	+0.25 -1.25 x 17	XT 25 PD	
**S**	12	RE	0.2	0.0	ABSENT	-1.50 × 150	+3.50 -2.50 × 25	ET 80 PD	
**S**	22	RE	0.5	0.0	ABSENT	+ 1.50 -1.00 × 170	+0.50 -0.75 × 175	ET 15 PD	
**S**	11	RE	0.5	- 0.1	ABSENT	+ 7.00 -1.00 × 15	+6.00 -1.00 × 170	ET 50 PD	
**S**	35	LE	0.0	0.4	400"	+ 1.50 -0.75 × 180	+1.75 -0.50 × 180	ET 10 PD	
**S**	13	RE	0.2	- 0.1	ABSENT	+ 1.50	+1.50	ET 30 PD DVD	
**S**	9	LE	0.2	0.4	ABSENT	+ 7.50 -1.50 × 165	+8.00 -1.00 × 10	ET40 PD E/D 6 PD	
**A**	30	LE	-0.2	0.1	100"	-0.75 × 90	-2.50 -1.75 × 80	ABSENT	NEGATIVE
**A**	12	LE	-0.1	0.3	80"	PLANE	+5.00 -2.50 × 10	ABSENT	NEGATIVE
**A**	14	LE	-0.1	0.1	200"	+ 2.25 -1.50 × 180	+3.50 - 3.75 × 170	ABSENT	NEGATIVE
**A**	10	RE	0.1	-0.1	50"	-9.00 - 4.00 × 10	+0.75 -1.00 × 180	ABSENT	NEGATIVE
**A**	39	LE	0.1	0.8	400"	+ 4.00 -1.00 × 30	+3.00 -2.00 × 180	ABSENT	POSITIVE
**A**	12	RE	0.1	-0.2	80"	-4.00 -0.50 × 130	-0.50 × 10	ABSENT	NEGAT1VE
**A**	8	RE	0.5	0.0	400"	-11.50 -2.75 × 20	-3.50 -0.75 × 180	ABSENT	NEGAT1VE
**A**	8	LE	0.0	0.7	400"	+ 0.75 -0.75 × 180	+5.25 -1.25 × 180	ABSENT	POSITIVE
**A**	12	RE	0.0	- 0.3	50"	+2.50	+0.25	ABSENT	POSITIVE
**A**	15	RE	0.1	- 0.2	400"	+3.00	+0.50	ABSENT	POSITIVE
**A**	8	RE	0.3	0.0	100"	-3.25 × 180	+1.50	ABSENT	POSITIVE
**A**	35	RE	0.8	- 0.1	ABSENT	+7.50	-0.50 -0.50 × 180	ABSENT	POSITIVE
**A**	15	LE	- 0.1	0.3	100"	-0.50 -0.50 × 10	+ 1.75 -1.25 × 175	ABSENT	POSITIVE
**A**	15	LE	- 0.2	0.1	100"	PLANO	+3.25 -0.50 × 40	ABSENT	POSITIVE
**A**	15	RE	0.1	- 0.2	50"	+ 2.00 -0.50 × 180	PLANO	ABSENT	POSITIVE
**A**	29	LE	0.0	0.4	3000"	-0.50 -1.25 × 160	-4.00 -1.75 × 170	ABSENT	POSITIVE
**A**	29	LE	-0.2	0.6	ABSENT	PLANO	+8.00	ABSENT	POSITIVE
**A**	14	LE	-0.1	0.6	200"	+ 1.50	+5.50	ABSENT	
**M**	13	LE	0.0	0.6	120"	+6.00 -2.00 × 160	+ 10.25 -3.00 10	MICRLET	POSITIVE
**M**	7	LE	0.0	1.0	100"	+2.00	+6.00	MICRLET	POSITIVE
**M**	10	RE	0.5	0.1	ABSENT	+5.50 -1.50 × 15	+3.75 -0.75 × 180	ET 50 PD	NEGATIVE
**M**	9	LE	-0.1	0.5	ABSENT	+ 1.75	-4.50	XT 30 PD	NEGATIVE
**M**	10	LE	0.0	0.3 P	ABSENT	+2.50 -1.50 × 180	+3.75 -4.00 × 180	ET 15 PD	NEGATIVE
**M**	10	LE	0.0	0.4	80"	+5.25 -0.50 × 20	+5.50 -2.00 × 170	MICRLET	POSITIVE
**M**	12	RE	0.7	-0.1	200"	-3.00	+0.50	XT 20 P	NEGATIVE
**M**	12	RE	0.4	0.0	ABSENT	-1.75 -1.25 × 90	-0.50 -1.25 × 90	XT 40 PD	NEGATIVE

A= anisometropic amblyopia; ET= esotropia; LE= left eye; M= mixed amblyopia; M= monofixation; PD= prismatic diopters; RE= right eye; RX= refraction; S= strabismus amblyopia; SVA= stereoscopic visual acuity (seconds of arc); VA= visual acuity (logMAR); XT= exotropia.

**Table 2:** Descriptive data of control participants in our study

Group	Age	Dominant	VA RE	VA LE	SVA	RX RE	RX LE	Deviation
C	15	LE	-0.1	0.0	40"	PLANE	PLANE	ABSENT
C	15	RE	0.0	0.0	40"	-0.25 -0.50 × 180	-0.50	ABSENT
C	17	RE	0.0	0.0 P	40"	-2.50 -0.50 × 180	-3.00 -0.50 × 180	ABSENT
C	11	LE	-0.2	-0.1	40"	PLANE	PLANE	ABSENT
C	24	LE	0.0	0.0	40"	-6.00	-6.00	ABSENT
C	36	LE	-0.2	-0.1	40"	-0.50 × 180	PLANE	ABSENT
C	15	RE	0.0	0.0 P	40"	PLANE	PLANE	ABSENT
C	17	LE	-0.1	-0.2	40"	-4.75 -0.50 × 180	-4.75 -0.50 × 180	ABSENT
C	10	RE	-0.1	-0.1	40"	-2.25	-1.25	ABSENT
C	7	LE	0.0	-0.1	40"	+0.75	+0.50	ABSENT
C	9	LE	-0.1	-0.1	40"	PLANE	PLANE	ABSENT
C	12	LE	-0.1	-0.1	40"	-2.00	-1.25	ABSENT
C	7	LE	0.1	0.0	40"	PLANE	PLANE	ABSENT
C	11	RE	-0.1	-0.1	40"	PLANE	+0.50	ABSENT
C	13	RE	0.0	-0.1	40"	+0.50	+0.25	ABSENT
C	9	RE	-0.1	-0.1	40"	PLANE	PLANE	ABSENT
C	11	RE	-0.1	0.0	40"	+2.75 - 1.25 × 180	+2.75 1.25 × 180	ABSENT
C	19	RE	-0.1	-0.1	40"	-3.00	-3.50	ABSENT
C	9	RE	-0.1	-0.1	40"	-4.00	-4.00	ABSENT
C	13	RE	0.0	0.0	40"	PLANE	-0.25	ABSENT
C	12	RE	-0.2	-0.2	40"	PLANE	PLANE	ABSENT
C	16	RE	-0.3	-0.3	40"	PLANE	PLANE	ABSENT
C	15	LE	-0.2	-0.2	40"	PLANE	PLANE	ABSENT
C	11	RE	-0.3	-0.3	40"	-0.50 × 180	-0.50 × 180	ABSENT
C	11	RE	-0.3	-0.3	40"	PLANE	PLANE	ABSENT
C	17	LE	-0.3	-0.2	40"	PLANE	PLANE	ABSENT
C	16	LE	-0.1	-0.1	40"	PLANE	PLANE	ABSENT
C	16	RE	-0.1	-0.1	40"	PLANE	PLANE	ABSENT
C	16	RE	-0.3	-0.2	40"	+ 0.75	+ 1.00	ABSENT
C	21	LE	-0.1	-0.1	40"	PLANE	PLANE	ABSENT
C	18	RE	0.0	0.1	40"	PLANE	-0.75 × 180	ABSENT
C	15	RE	-0.1	-0.1	40"	PLANE	PLANE	ABSENT
C	9	RE	-0.1	-0.1	40"	-3.50	-3.50	ABSENT

C= control; LE= left eye; RE= right eye; RX= refraction; SVA= stereoscopic visual acuity (seconds of arc); VA= visual acuity (logMAR).

Severe amblyopia was found in 16% of the amblyopic individuals, with 4.76% of these with strabismus, 7.14% with anisometropia and 4.76% with the mixed type.

In the controls, the mean VA of the DE was -0.096 logMAR; that of the NDE was -0.098 logMAR. In the amblyopic groups, the mean VA was -0.067 logMAR for the DE and 0.349 logMAR for the NDE (Table 3).

**Table 3:** The mean visual acuity values of our control group and amblyopia group in our study and statistical comparisons of the two groups

	AVERAGE VA (logMAR)			p-value
	Control	Amblyopia	RM-ANOVA	
Dominant eye	-0.096	-0.067	F = 4.88*	0.030
Non-dominant eye	-0.098	0.349	F = 78.61*	<0.001

RM-ANOVA= repeated measures analysis of variance; VA= visual acuity.

An RM-ANOVA showed differences between the DE control and the amblyopic DE for VA (F = 5.83, p<0.001) and the NDE control and AE (F = 51.01, p<0.001). The interocular difference in VA was statistically different only between the NDE of the controls and the AE (F=119.41, p<0.001). The effect size for these measurements was very large (η² = 0.821).

The DEs and NDEs of the control group were compared to each amblyopia type group (strabismus, anisometropia, and mixed) for VA. The mean VA of the DE was -0.09 logMAR (0.11 SD) in the control group, -0.04 logMAR (0.08 SD) in the strabismus group, -0.11 logMAR (0.10 SD) in the anisometrope group, and -0.01 logMAR (0.06 SD) in the mixed group. For the NDE, the mean VA was -0.09 logMAR (0.11 SD) in the control group, 0.40 logMAR (0.18 SD) in the strabismus group, 0.33 logMAR (0.27 SD) in the anisometrope group, and 0.55 logMAR (0.21 SD) in the mixed group.

**Figure 2:**
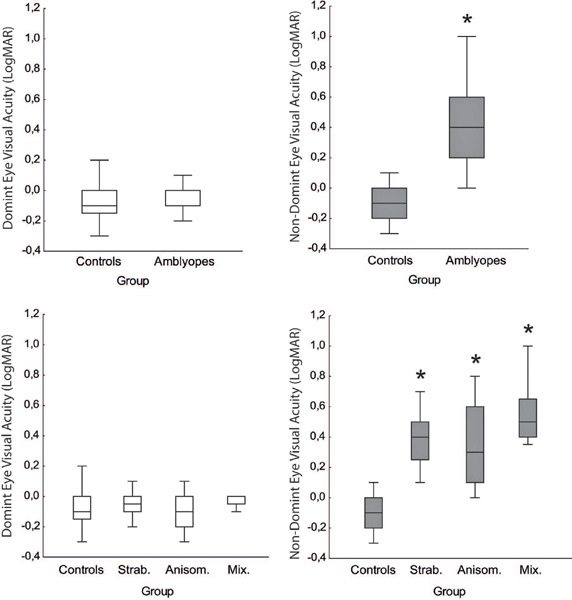
Comparison of the mean visual acuity (VA) of our control group and three amblyopia type groups for the dominant eye (left) and the non-dominant (right). Mean (o) and confidence interval (CI).

There was a significant difference between the VA of the AEs compared to the VA of the DEs (F = 3.95, p = 0.012) and NDEs (F = 33.10, p<0.001). Post hoc analysis showed significant differences between the DEs of the control group and the strabismus group and the control group and anisometropic group. For the NDE, there were significant differences between the controls and all types of amblyopia. The mixed group also differed significantly from the strabismus and anisometrope groups (Figure 2).

**Figure 3:**
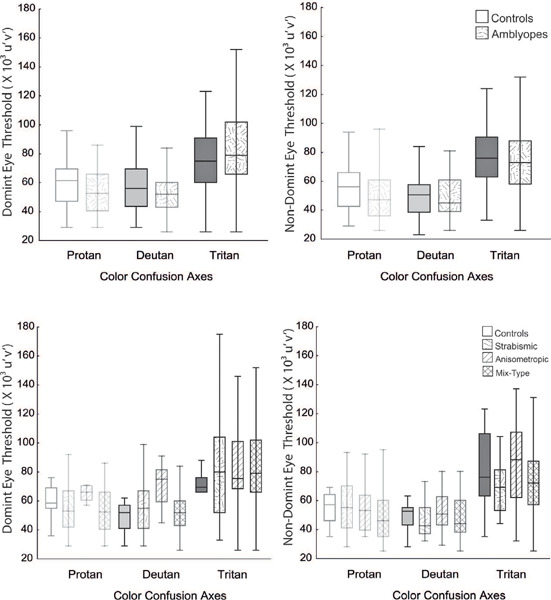
Comparison of the chromaticity threshold measurements obtained on the protan, deutan, and tritan axes of the control group and the three amblyopia type groups for the dominant and non-dominant eye.

Color vision was analyzed in our sample by comparing the chromaticity thresholds of participants with measurements of the protan, deutan, and tritan color confusion axes. There was no statistically significant difference in chromaticity discrimination between amblyopic patients and controls (p>0.05) (Figure 3). This remained the case when each amblyopic type group was separately compared to the control group. There were no statistically significant difference (p>0.05) between the control groups and amblyopic type groups in the mean chromaticity thresholds on the protan, deutan, and tritan axes (Figure 3). No differences were found between the DE and NDE within the control group or within the amblyopic groups.

We found a moderate inverse correlation between the age and chromaticity threshold values for the DE (protan, r-0.46; deutan, r-0.42; tritan, r-0.41) and NDE (protan, r-0.47; deutan, r-0.49; tritan, not significant) of the control group. However, there were no significant correlations in any of the amblyopia groups. No correlation was found between the VA results and the chromaticity thresholds of either controls or amblyopic participants.

## DISCUSSION

We evaluated color vision through chromaticity discrimination without shape, luminance, and contours in the visual field to provide spatial cues. This was achieved using a pseudoisochromatic arrangement highly controlled for component sizes and luminance^(15)^. As previous studies using this method have shown that the spatial variables involved in early visual processing do not affect chromaticity discrimination, we hypothesized that amblyopic patients would have no color discrimination deficits. This hypothesis was confirmed as there was no difference in color discrimination on any of the three color confusion axes between patients with residual amblyopia of different etiologies and age-matched control participants. Likewise, no correlation was found between color discrimination sensitivity and VA in these patients, suggesting independent processing of color discrimination information via the PC pathway. We also found an inverse correlation in the control group between age and chromaticity threshold values for both the DE and NDE. This implies a disorganized pattern of spatial feature development within the visual system^(23^,^24)^.

Color vision is an understudied visual function in amblyopia. This study makes an important contribution to the topic through its identification of differences in the visual processing of space and color. VA is known to be dependent on the visual processing that occurs in the central area of the retina and via the PC visual pathway. Developmental changes in VA in amblyopia suggest that the condition may also affect chromatic visual processing^(25)^. However, our data suggest parallel processing of space and color. This was evidenced through the use of isolated evaluation of chromatic discrimination with no spatial cues in the visual field. This allows the parallel processing of space and color to be preserved up to primary visual cortex. Based on this evidence for different visual processing mechanisms for space and color, it could also be posited that amblyopia treatment would not affect color vision in contexts lacking spatial components such as the visual stimuli used in the CCT. However, one would expect changes in sensitivity to chromatic contrast when the color is spatially distributed. This idea was corroborated by our finding that there is no correlation between VA and chromaticity threshold. This again suggests that spatial features of the visual field are processed separately from chromaticity information. Previous studies have found decreased chromatic CS in strabismic amblyopia, with greater deficits in chromatic CS than luminance CS, suggesting that the condition exerts greater effects on the PC pathway than the magnocellular (MC) pathway, which is achromatic^(12)^. Using chromatic and luminance grating stimuli, Mullen et al. have demonstrated a greater deficit in positional estimation accuracy with chromatic than achromatic stimuli in amblyopic patients^(11)^. These authors suggest that both the chromatic (PC and KC) and achromatic (MC) pathways are affected by amblyopia, but that the effects on chromatic vision are greater, as demonstrated by the lower fidelity of chromatic than achromatic spatial representation. There is some discrepancy between these studies and our own regarding the control of spatial components. Spatial components are frequently incorporated into stimuli used to measure CS. These include periodical sine or square waves and checkerboards. In such stimuli, the spatial components can be considered the dominant variables since the evaluation is made by using the dispersion of luminance, wavelength, or chromaticity over the space. Because we evaluated chromaticity CS without spatial cues, the lack of color-vision impairment findings in our amblyopic participants supports our hypothesis that spatial aspects of the visual field are the most affected by this condition. Considering this, it is important that clinicians understand the nature of the color test used in this study before applying it in clinical practice since the results may differ greatly depending on the presence or absence of spatial, temporal, or positional components in the stimuli images. These must be excluded for accurate testing of the aspects of vision concerned with hue, saturation, or chromaticity.

This study also contributes to the body of research into different types of amblyopia, which are distinguished by etiology. We have shown that there are similar color-vision discrimination thresholds between normal DEs and AEs, regardless of disease etiology. The current understanding is that there are clinically important differences between the visual performance of children with strabismic amblyopia and those with anisometropic. Those with strabismic amblyopia have worse binocular vision but better VA than those with anisometropic amblyopia. However, these differences were not supported by our results from evaluations in which the measurements are of purely chromatic features. The thresholds for the protanopic and deuteranopic confusion axes, both of which are processed by the PC pathway, did not differ significantly from those of the control group. The tritanopic axis, processed through the KC pathway, was also unaltered by amblyopia. This discrepancy between our findings and those of previous studies is likely due to stimulus construction differences.

We evaluated our participants using the CCT. This assesses chromaticity discrimination on the three confusion axes in a color space that reproduces the experience of chromaticity produced by retinal input (CIE 1976 u’v’). It presents a pseudoisochromatic stimulus that eliminates shape, luminance, and simultaneous contrast cues using the base pattern configuration and a luminance noise. The test provides a more efficient presentation of chromaticity levels and changes, more refined levels, and more stimulation possibilities than standard printed pseudoisochromatic plates^(13)^. In a previous study, we found no ocular dominance or binocular summation effects on chromaticity discrimination. This supports the supposition that spatial components do not interfere with chromaticity discrimination when a pseudoisochromatic stimulus arrangement is used. Our findings agree with those of previous studies that have failed to find changes in color discrimination in amblyo-pia^(8^,^9)^. This result suggests that color discrimination is dominated by functional processing at early levels (the retina and possibly the primary visual pathway) and is spared in amblyopia, regardless of etiological type.

Conversely, another previous work found changes in chromatic processing in amblyopia using measures of chromatic CS^(11)^. This used standardized periodic stimuli (sinusoidal grids and checkerboards) with an intrinsic spatial component linked to the color stimulus. Based on the physiological responses identified in the primary and secondary visual cortices, it can be inferred that these are processed in different cortical areas. Visual areas of the V2 known as thin streaks receive their input from V1 blob cells and are therefore related exclusively to chromatic processing exclusively. However, the areas of V2 between stripes primarily process spatial aspects of vision related to the passage of the PC pathways into the V1^(26)^. There are many intraneuronal connections between these two areas. This allows chromatic stimuli with spatial components to change in ways that pure chromatic stimuli cannot. Thus, the retinal and cortical discrimination apparatus are maintained in amblyopia; although, the spatial integration of color information is not. The only work to date to study color vision in children with low VA (including those with amblyopia) using CCT found no correlation between VA and color discrimination sensitivity^(16)^. Our results are in concordance with this finding. This strongly supports the above assertion that only the spatial components of color discrimination are defective in amblyopia. Thus, we suggest that further research into color vision in amblyopia should utilize stimuli that require the integration of color information with visual spatial data to determine the impact on the PC and KC pathways. This will provide comparative results that can be considered alongside the present findings from our assessment of color discrimination capability as an isolated visual function. Although our amblyopic participants had chromatic discrimination thresholds comparable to those of visually healthy controls, there correlation measures was a moderate inverse correlation between age and chromaticity threshold in both the DEs and NDEs of the control group. This correlation has previously been demonstrated by Knoblauch et al.^(24)^. The absence of these correlations in our amblyopia group makes it difficult to ascertain whether there are any systematic changes in threshold with age in these patients. Alterations in the color-vision developmental curve in amblyopic individuals cannot be inferred from our findings as this was not a longitudinal study. Further research is needed to investigate this possibility.

Finally, our results support the recent conceptualization of amblyopia as a visual alteration of neurological origin that affects various anatomical structures involved in visual processing and exerts effects on several processing levels. Despite centuries of study of this visual disease, our findings highlight the gaps in our knowledge of its pathophysiology and the many unanswered questions about the condition^(1)^. For clinicians, answers to these questions will facilitate understanding, optimize management of the residual visual consequences, and improve preventive measures and treatment of amblyopia. For researchers, our findings highlight the need for further studies of amblyopia that increase our understanding of the mechanisms, the evolution of classifications, and the diagnostic methods and criteria.

In this study, we have shown that there is no change in color discrimination in residual amblyopia when color stimuli have no spatial components. This is a new finding since most chromatic CS tests evaluate this characteristic using colors distributed over the visual space. Our findings suggest that some aspects of PC and KC processing are affected by amblyopia, especially those involving spatial processing.
